# A Comprehensive Comparison of LRYGB and LSG in Obese Patients Including the Effects on QoL, Comorbidities, Weight Loss, and Complications: a Systematic Review and Meta-Analysis

**DOI:** 10.1007/s11695-019-04306-4

**Published:** 2019-12-13

**Authors:** Zhihao Hu, Junfeng Sun, Ruixin Li, Zhuoyin Wang, Hengxuan Ding, Tianyu Zhu, Guojun Wang

**Affiliations:** Department of Gastrointestinal Surgery, The First Affiliated Hospital of Zhengzhou University, NO.1 Jianshe East Road, Zhengzhou, 450052 Henan China

**Keywords:** Laparoscopic Roux-en-Y gastric bypass, Laparoscopic sleeve gastrectomy, Three-stage analysis, Obesity surgery

## Abstract

**Purpose:**

To systematically and comprehensively evaluate the differences between laparoscopic Roux-en-Y gastric bypass (LRYGB) versus sleeve gastrectomy (LSG) in obese patients.

**Methods:**

A systematic literature search was performed in PubMed, EMBASE, Web of Science, and the Cochrane Library from inception to December 2018. The meta-analysis was performed by the RevMan 5.3 software.

**Results:**

Twenty-three articles with 7443 patients were included. In short term (< 3 years), LRYGB was superior to LSG in terms of improving comorbidities (T2D, odds ratio (OR) 1.93, 1.06–3.52, *P* < 0.05, hypertension, OR 1.59, 1.08–2.34, *P* < 0.05, dyslipidemia, OR 1.61, 1.05–2.46, *P* < 0.05), but there were no differences in the midterm and long term. Quality of life (QoL) after bariatric surgery was included, but no differences were observed in the QoL after LRYGB or LSG (gastrointestinal quality of life index (GIQLI) and Moorehead–Ardelt quality of life questionnaire (M-A-Q), *P* > 0.05). LRYGB achieved a higher EWL% than LSG (after 3 years, WMD 5.48, 0.13–10.84. *P* < 0.05; after 5 years, WMD 4.55, 1.04–8.05, *P* < 0.05) in long term, but no significant differences were found during 0.25- to 2.0-year follow-up. The rate of early and late complications was much higher in LRYGB than in LSG (early complications, OR = 2.11, 95% CI = 1.53–2.91, *P* < 0.001; late complications, OR = 2.60, 95% CI = 1.93–3.49, *P* < 0.001).

**Conclusions:**

This meta-analysis showed that LRYGB was more effective than LSG in comorbidities’ resolution or improvement in short term. For weight loss, LRYGB had better long-term effects than LSG. In addition, no differences were observed in the quality of life after LRYGB or LSG. LRYGB was associated with more complications than LSG.

**Electronic supplementary material:**

The online version of this article (10.1007/s11695-019-04306-4) contains supplementary material, which is available to authorized users.

## Introduction

With its increasing prevalence, obesity has become a global public health problem over the past few decades [1]. Being overweight is accepted as a risk factor for serious health issues, such as type 2 diabetes, hypertension, diseases, or even cancers [2–4]. Compared with various strategies, including medications, behavior changes, and diet therapy, bariatric surgery is still recognized as the most effective treatment for weight loss and improvements of the associated comorbidities [5–7].

Primary bariatric procedures include Roux-en-Y gastric bypass (RYGB), laparoscopic adjustable gastric banding (AGB), laparoscopic vertical banded gastroplasty (VBG), sleeve gastrectomy (SG), mini-gastric bypass/one anastomosis gastric bypass (MGB/OAGB), biliopancreatic diversion/duodenal switch (BPD/DS), and single-anastomosis duodeno-ileal bypass (SADI). Among these techniques, laparoscopic Roux-en-Y gastric bypass (LRYGB) and laparoscopic sleeve gastrectomy (LSG) have gained the most popularity. However, there is a sharp trend towards the utilization of LSG over the last decade and a decline in the use of LRYGB [8, 9]. Some studies suggest that LSG is easier and faster to perform and potentially safer compared with LRYGB [8, 10], while some indicate that LRYGB is more potent than LSG [11–13].

Some meta-analyses of LRYGB versus LSG have been performed before [14–17], but obvious shortcomings remain. Of the previous studies, some lack adequate stratified analysis with respect to EWL% and comorbidities [14, 15].

Here, we performed a comprehensive meta-analysis comparing LRYGB with LSG with respect to their early and late complications, and amount of weight loss at different time points after surgery, as well as the effect on comorbidities at three different terms (short term, midterm, and long term) and quality of life (presented by GIQLI and M-A-Q II).

## Materials and Methods

We performed a comprehensive literature search in PubMed, EMBASE, Web of Science, and the Cochrane Library from inception to December 2018. Our search strategy included the following key terms: laparoscopic sleeve gastrectomy, LSG, SG, LRYGB, RYGB, bariatric surgery, and obesity. The reference lists of potential articles as well as the extraction data were screened manually by two independent reviewers (Hu and Sun). Any data extraction inconsistency was assessed by a third reviewer (Li).

Inclusion criteria are as follows: (1) sample size of every group > 15 patients; (2) human study reported in English; (3) at least one of the following endpoints was included: early complications, resolution rate of comorbidities, and weight loss (performed as EWL%); (4) patient ages ranged from 18 to 70 years old; (5) comparative studies between LRYGB and LSG. Exclusion criteria are as follows: (1) non-human studies; (2) non-laparoscopic surgery; (3) studies that only included LRYGB or LSG; (4) case reports, analyses, comments, and overviews.

### Definition of Endpoints

Early complications were defined as those occurring within 30 days after surgery, while late complications occurred over 30 days. For resolution or improvement rate of comorbidities, the definitions of different terms were as follows: short term (1 year after surgery), midterm (3 years), long term (5 years).

### Data Extraction

The following data were independently extracted from each eligible study: author, publication year, study design, sample size, overall rate of early and late complications, resolution/improvement rate of comorbidities (T2DM, hypertension, OSA, dyslipidemia), and weight loss at every follow-up point. The extraction was completed by two reviewers (Hu and Sun).

### Statistical Analysis

Review Manager for the Windows version 5.3 (The Nordic Cochrane Centre, Copenhagen, Denmark) was used for analysis. Weighted mean differences (WMD) with 95% confidence intervals (CI) were used to analyze continuous data, while odds ratios (ORs) with 95% CIs were used for the statistical analyses of dichotomous data. Heterogeneity was represented by I2 (low heterogeneity at values< 30%, moderate heterogeneity at values 30–50%, and high heterogeneity at values > 50%). The random-effects model was used for the analysis of studies with high heterogeneity, and the fixed-effects model was used for studies with low or moderate heterogeneity.

### Quality and Publication Bias Assessment

The methodological quality of the included non-RCT studies was determined by the NOS (Newcastle–Ottawa scale). When the study scored ≤ 5, the study was assessed as low quality; when the study scored > 5, the study was assessed as high quality and was included in our meta-analysis. The methodological quality of the included RCTs was determined using Cochrane Collaboration’s tool for risk of bias. Sensitivity analyses were performed by removing individual studies from the whole set of studies and analyzing the sources of significant heterogeneity. The exclusion of these studies did not influence the results. The funnel plot was used to measure publication bias. The shape of the funnel plot did not reveal obvious asymmetry (not shown).

## Results

**Fig. 1:**
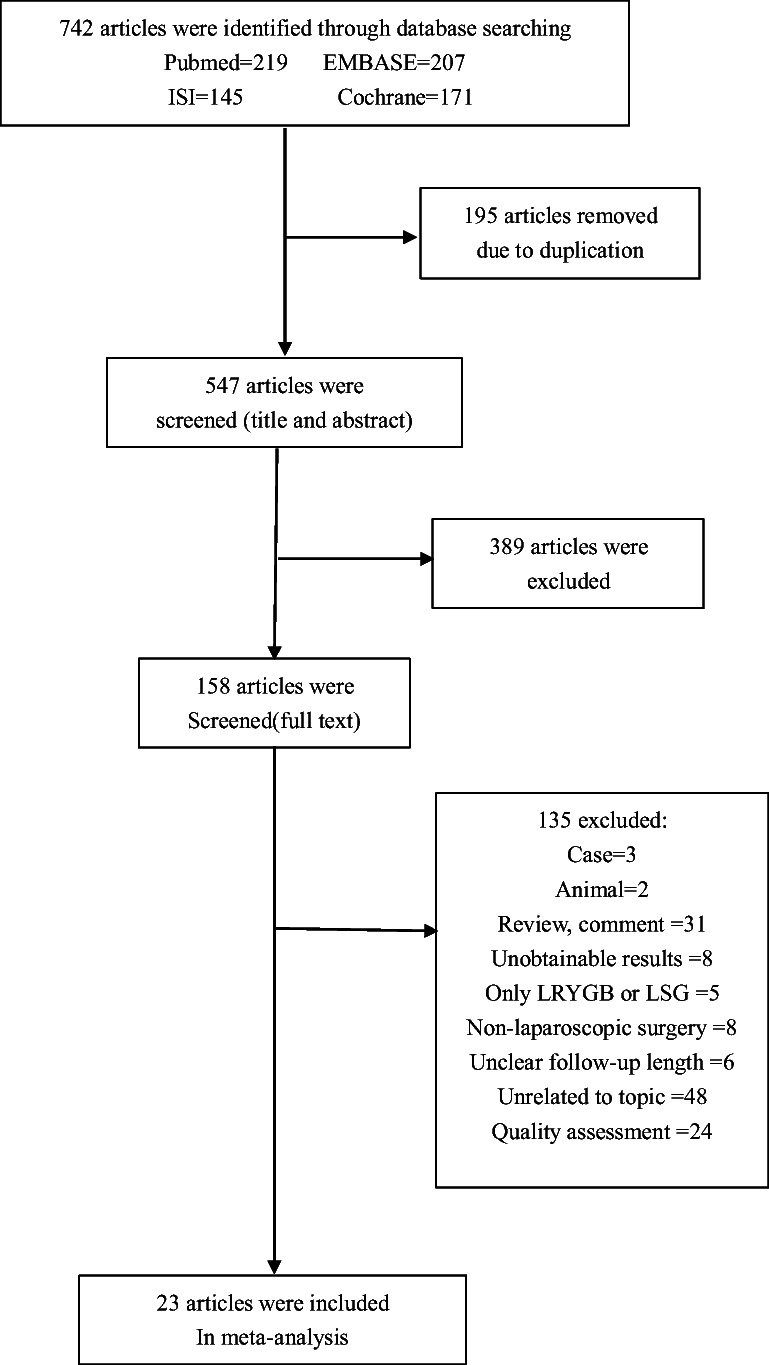
PRISMA flow diagram

The PRISMA flow diagram of our literature search is shown in Fig. 1. A total of 742 articles were identified from the database, of which 719 studies were excluded after duplication, text screening, and discussion. Finally, 23 articles [18–40] were included in our analysis; 9 of them are RCTs, 5 of them are prospective studies, and 9 of them are retrospective studies. In addition, three articles [30–32] were from the same RCT but were published at different times, as well as another two articles [34, 39]. Therefore, we combined them together in the following tables. The risk of bias for the RCTs is presented in Fig. S1. The characteristics of the included studies are shown in Table. S1.

### Complications

A total of 13 studies [18–20, 22, 24, 26, 27, 30, 33, 35, 37, 39, 40] reported early complications within 30 days, of which 5 studies [18, 20, 22, 26, 34] reported late complications. Overall, early complications occurred significantly more often after LRYGB than after LSG (OR = 2.11, 95% CI = 1.53–2.91, *P* < 0.001). As the definitions of major or minor complications were not consistent between studies, we did not perform the analysis of major and minor complications separately. Besides, LRYGB was also associated with more late complications than LSG (OR = 2.60, 95% CI = 1.93–3.49, *P* < 0.001).

**Fig. 2:**
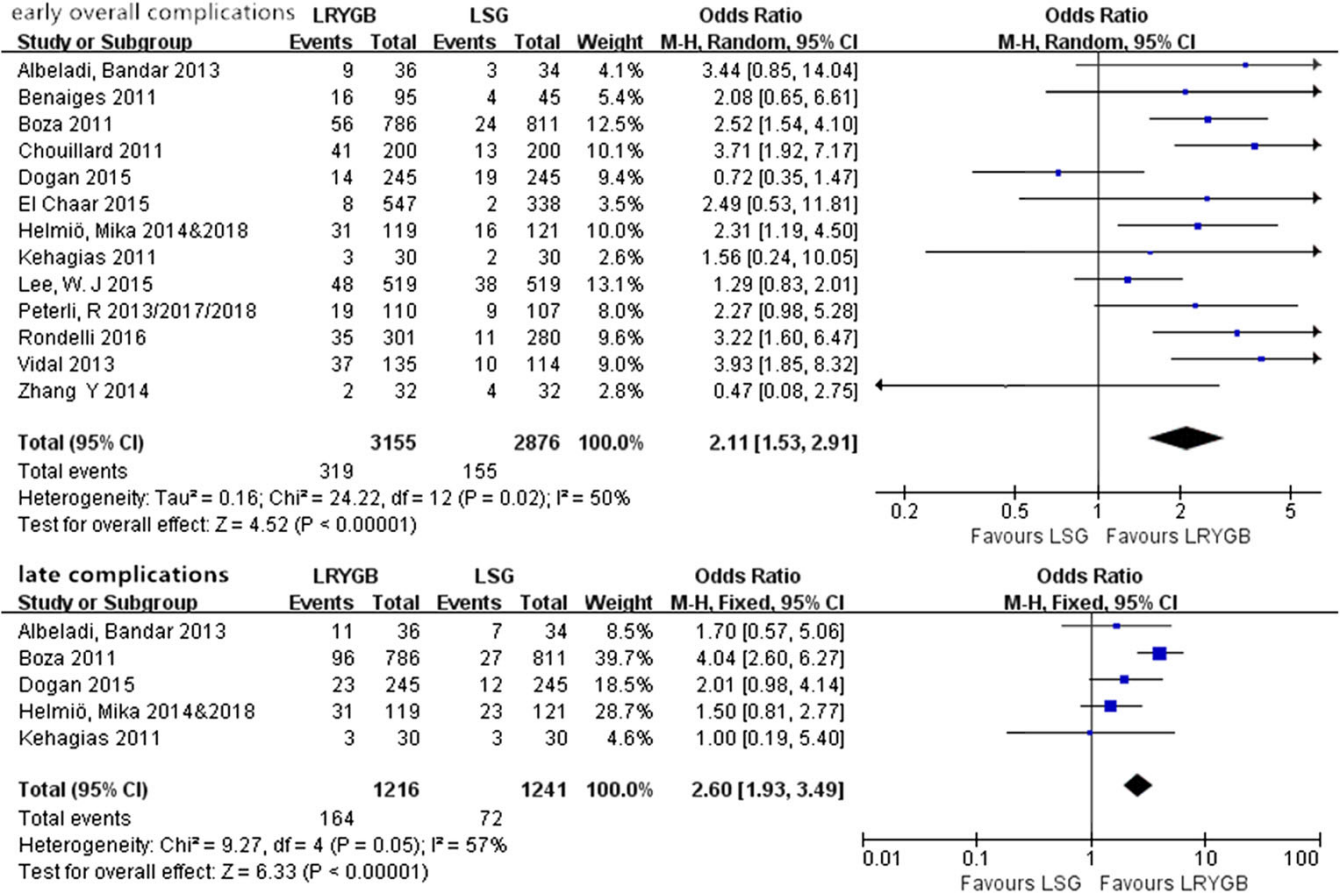
Complications after surgery

All of the above data are shown in Fig. 2.

### Weight Loss Outcomes (EWL%)

Most outcomes of weight loss are measured by EWL% ± SD, and we extracted data in our studies at 6 time points after surgery, including 3 months, 6 months, 1 year, 2 years, 3 years, and 5 years. There was high heterogeneity from 3 months to 3 years, and a random-effects model was applied to the analyses.

**Fig. 3:**
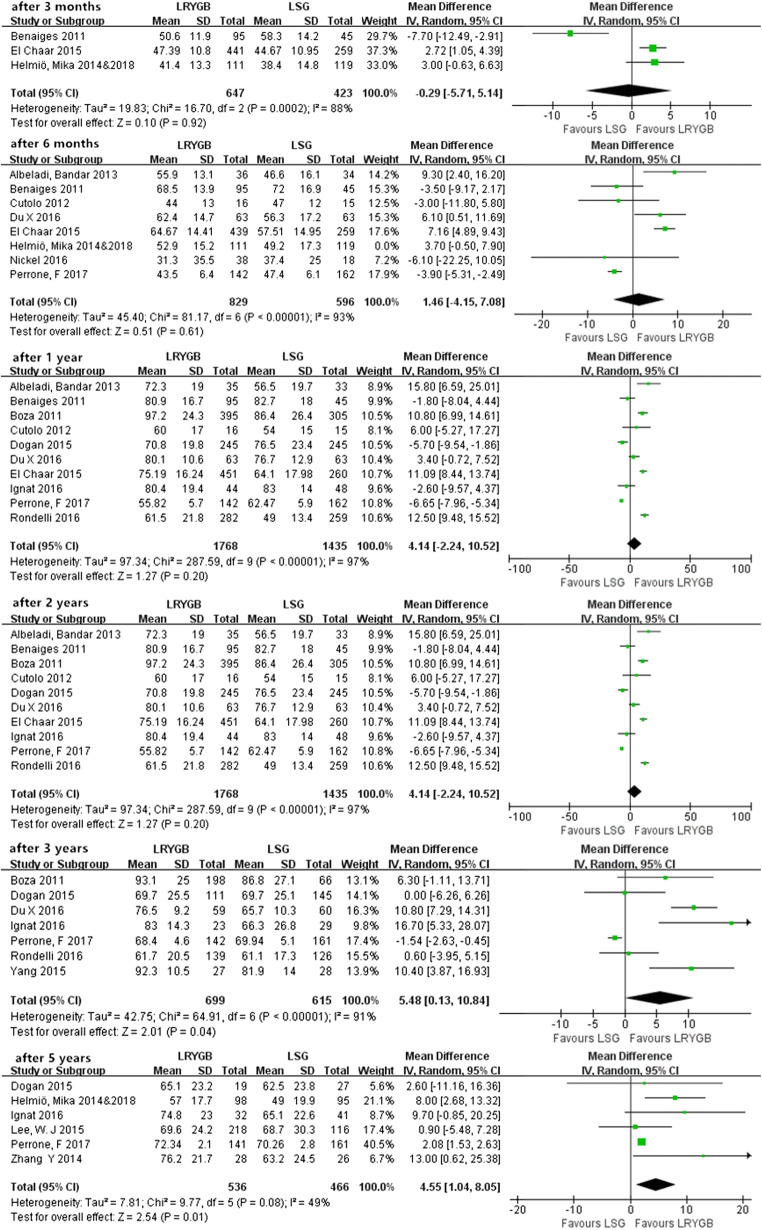
EWL% after 3 months, 6 months, 1 year, 2 years, 3 years, and 5 years

As shown in Fig. 3, there was no significant difference in the short term (from 3 months to 2 years) after surgery (*P* > 0.05). In contrast, in the midterm (after 3 years) and long term (after 5 years), LRYGB achieved a superior EWL% compared with LSG (*P* < 0.01).

### Resolution/Improvement of Obesity-Related Comorbidities

Several studies have researched the resolution/improvement rate of comorbidities. Considering that the rates of the comorbidities may differ at different times after surgery, we performed a meta-analysis at three different times.

#### Short-Term Obesity-Related Comorbidities

**Fig. 4:**
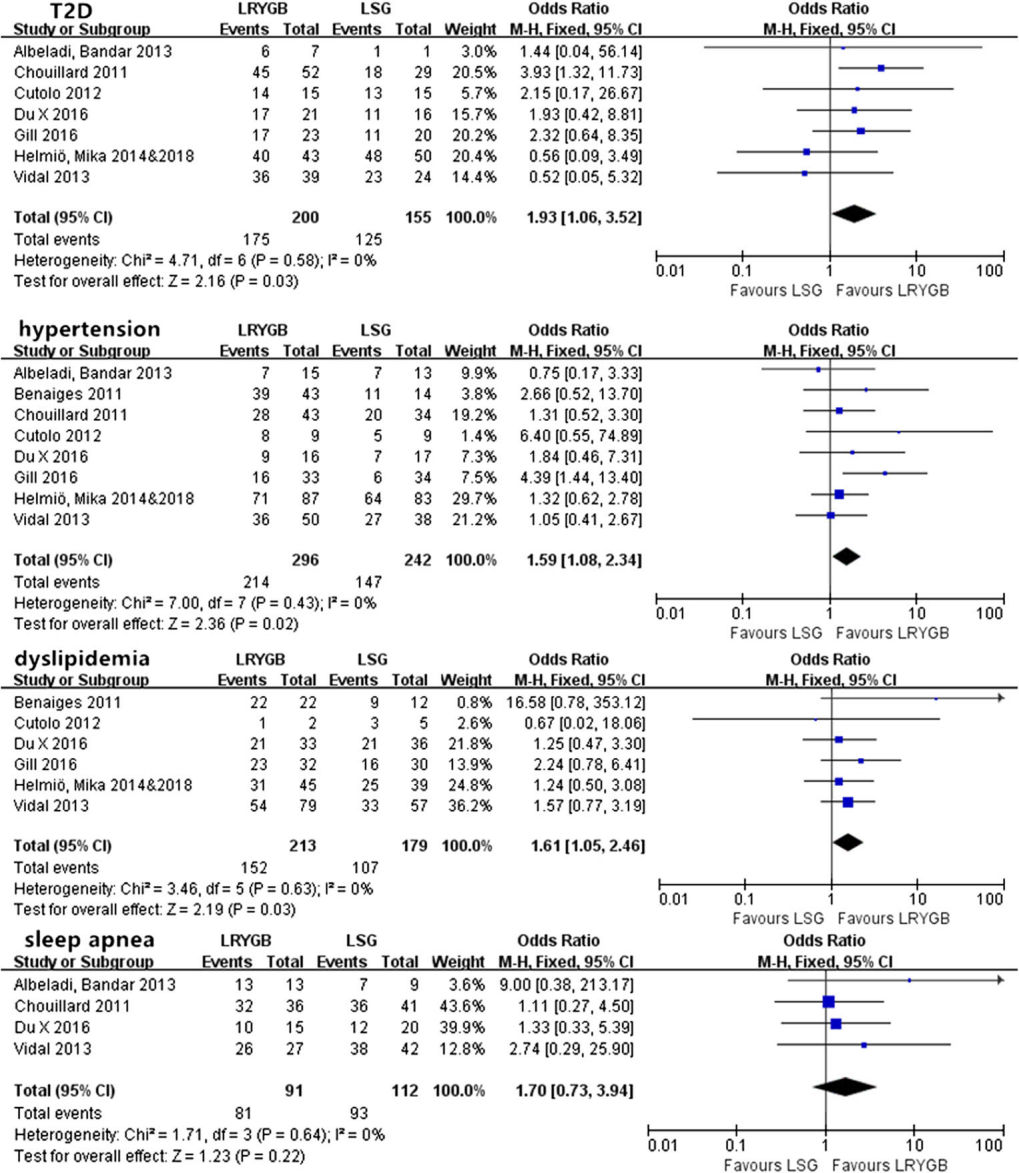
Short-term resolution/improvement rate

There was no remarkable heterogeneity in any of the comorbidities (*P* > 0.05), so fixed-effects models were used. Except for sleep apnea, we found that the resolution/improvement rate of T2D, hypertension, and dyslipidemia all showed significant differences between LRYGB and LSG (Fig. 4). In addition, LRYGB achieved a superior rate of resolution/improvement for T2D, hypertension, and dyslipidemia compared with LSG.

#### Midterm Obesity-Related Comorbidities

**Fig. 5:**
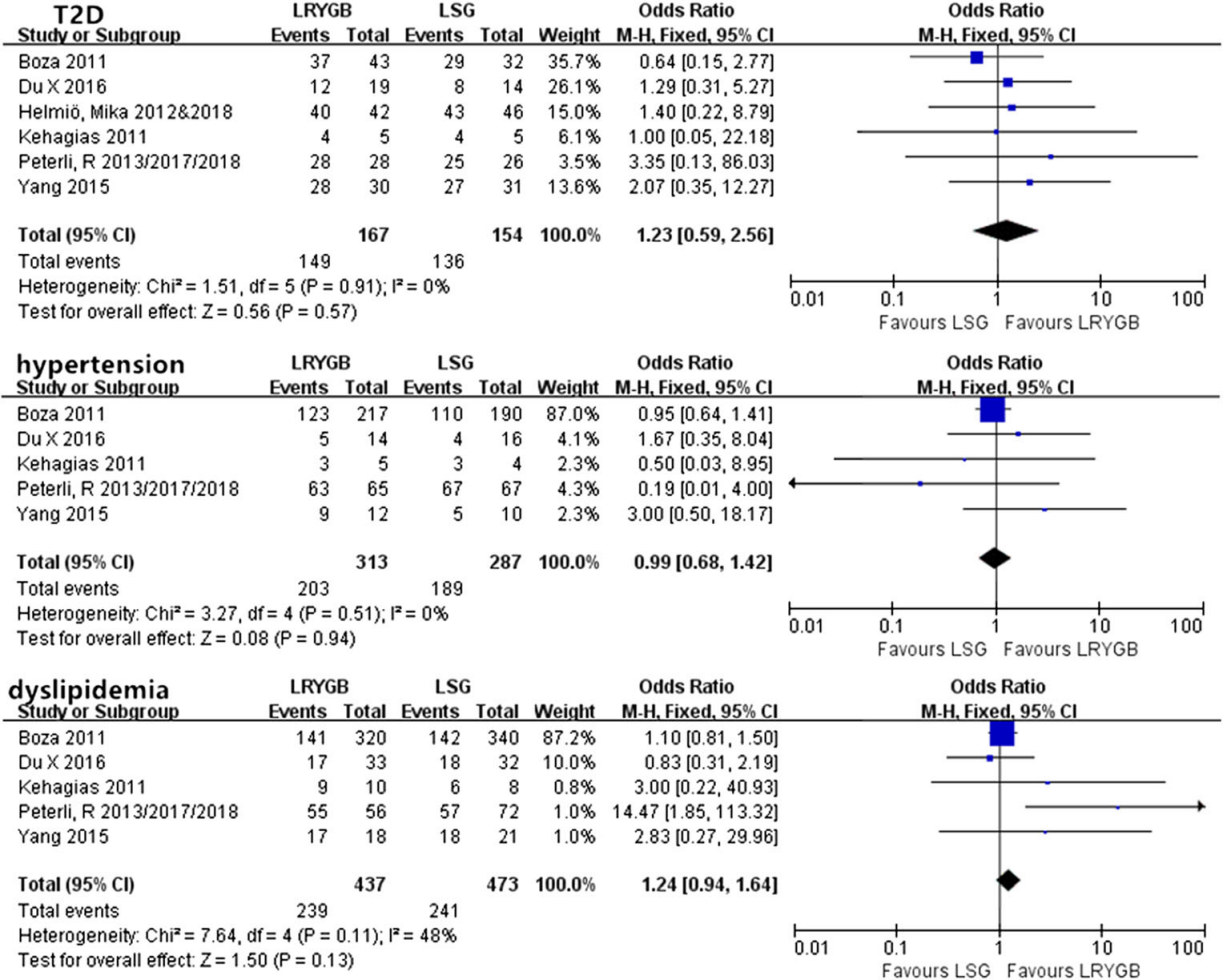
Midterm resolution/improvement rate

As shown in (Fig. 5), there were no significant differences in any of the comorbidities mentioned.

#### Long-Term Obesity-Related Comorbidities

**Fig. 6:**
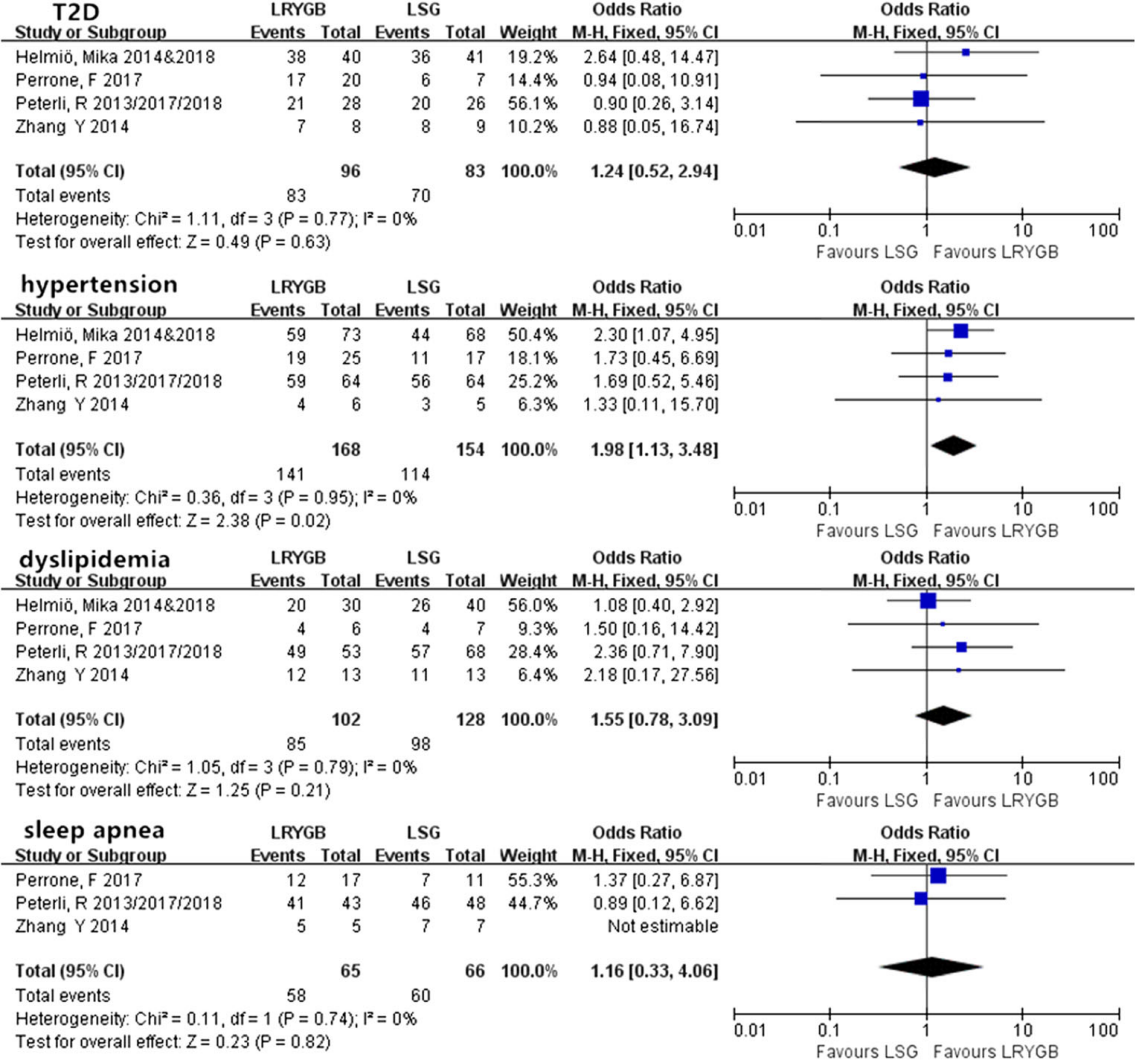
Long-term resolution/improvement rate

LRYGB achieved a better long-term (> 5 years) prognosis for hypertension after surgery than LSG with a significant difference (Fig. 6) (OR = 1.98, 95% CI = 1.13–3.48, *P* < 0.05). The other comorbidities showed no differences between the two procedures.

#### Quality of Life (GIQLI and M-A-Q)

The gastrointestinal quality of life index (GIQLI) is a questionnaire for gastrointestinal disease and includes 36 items that assess the following 5 aspects of life: core symptoms, physical items, psychological items, social items, and disease-specific items. The score of the questionnaire ranged from 0 to 144.

The Moorehead–Ardelt quality of life questionnaire II (M-A-QoL Q II) was designed to measure postoperative outcomes of self-perceived QoL in obese patients, including six parts: social relationship, self-esteem, physical activity, satisfaction concerning work, sexuality, and eating behaviors, with a total score that ranges from − 3.0 to + 3.0.

**Fig. 7:**
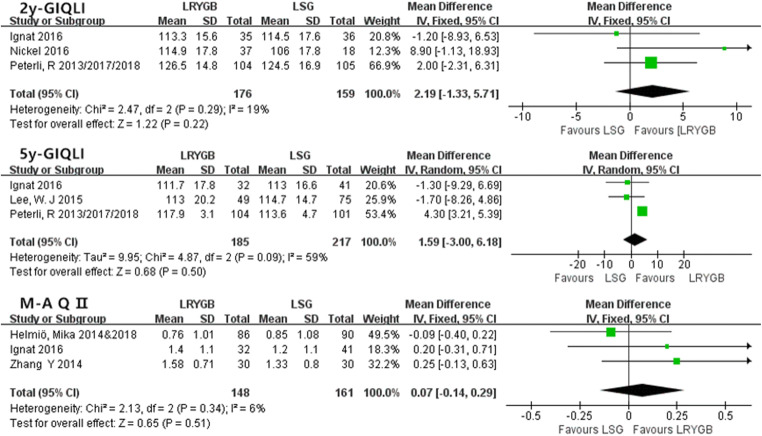
Quality of life

For GIQLI, no significant difference was observed between the two procedures after 2 and 5 years (at 2 years, WMD = 2.19, 95% CI − 1.33–5.71, *P* > 0.05 and at 5 years, WMD = 1.59, 95% CI − 3–6.18, *P* > 0.05). For the M-A-Q, there was also no difference between the outcomes (WMD = 0.07, 95% CI − 0.14–0.29, *P* > 0.05) (Fig. 7).

## Discussion

This research includes 23 studies with 3863 patients in the LSG group and 3580 patients in the LRYGB group. There have been some previous meta-analyses comparing LRYGB with LSG; however, this study is the first to systematically and comprehensively assess and compare the differences between the two surgical procedures from aspects of weight loss, rate of complications, the resolution/improvement rate of comorbidities, and quality of life in a single article.

### Complications

Regarding complications, LRYGB was significantly associated with more early complications than LSG, and the same result was observed for the late complications, which may be related to the difficulty of the LRYGB surgery. Compared with Osland’s study [41], we did not perform stratified analyses as the definitions of major or minor complications were not consistent between studies.

### Weight Loss Outcomes

In our research, we analyzed EWL% at different time points after operation. Li [14] and Zhang [15] have previously analyzed the outcomes of weight loss. However, Li did not perform stratified analyses according to time points, and his conclusion is unreliable, as the weight loss differs between time points. Although Zhang performed stratified analyses, the standard of data extraction was different from ours. As a result, there were some differences in our conclusion: we found that there was no significant difference in EWL% between the two surgical procedures during 0.25–2.0-year follow-up, but in the midterm and long term (3 years and 5 years, respectively), the LRYGB group had better effects than the LSG group in weight loss; this finding differs between our study and other studies. In addition, we find that the BMI in eastern country is lower than western country, which may lead to an inherent risk. But as the small number of eastern researches, a further analysis is necessary in future.

### Comorbidities

In terms of comorbidities, the resolution/improvement rates differ in different periods, but previous studies from Li [14] and Zhang [15] did not analyze the comorbidities according to time points, which led to the conclusion that LRYGB is better than LSG. The study from Shoar [42] included the midterm and long-term stages, but there was no short-term stage; additionally, the small number of inclusions was also mentioned as a limitation in Shoar’s study. For the first time, we introduced short-term studies and analyzed the midterm and long-term results at the same time. Our results showed that in the short term, LRYGB was superior to LSG in almost all aspects (except for sleep apnea because the sample size was not large enough). After including recent studies [29, 31, 34] of long-term follow-ups, the results showed that there were no differences between the two groups except for hypertension in the midterm and long term. Furthermore, over a longer time frame, the effect on comorbidities is equal to LSG despite better weight loss. The medical therapy may account for the result but we still need more researches.

### Quality of Life

This is the first time that quality of life after these two surgeries was summarized and analyzed with a meta-analysis. With GIQLI, we studied the scores 2 years and 5 years after the operation, and the results showed no obvious differences. For M-A-Q II, there was only sufficient data in the fifth year, but there was also no difference. However, we found that Nickel’s study [28] reported that the scores in the LRYGB group were significantly lower in the early period (within 6 months) than those in the LSG group. Combined with the results above, these outcomes may be related to the early complications. As LRYGB leads to more early complications, patients might focus on the difficult recovery in the early stages, but in the long term, the difference between the two groups decreases after recovery.

Our study has several limitations. Some of the included studies had a small sample size, which may affect the accuracy of this meta-analysis. Re-using a selection of 3–10 studies from the pool of 23 articles for each different research question may lead to the inherent risk of missing relevant publications for each of the individual forest plots. In terms of weight loss, the heterogeneity of analysis at each time point was high, which may be related to different race and region, and different surgical level. Besides, we only searched for English literature; therefore, language bias might exist in this research.

## Conclusion

In this meta-analysis, we found that LRYGB was superior to LSG for comorbidities resolution/improvement in short term, with no difference in the midterm and long term. There was no significant difference in weight loss between LRYGB and LSG in the early period, but LRYGB showed better long-term outcomes in weight loss; in addition, no differences were observed in the quality of life after LRYGB or LSG. The rate of complications was higher for LRYGB than for LSG.

## Electronic Supplementary Material

ESM 1 (DOCX 18 kb)

ESM 2 (PNG 7 kb)
