# Supplementary material for: A Comprehensive Comparison of LRYGB and LSG in Obese Patients Including the Effects on QoL, Comorbidities, Weight Loss, and Complications: a Systematic Review and Meta-Analysis
Source: Obes Surg. 2019 Dec 13;30(3):819–27. doi: 10.1007/s11695-019-04306-4 (PMC7347514; doi:10.1007/s11695-019-04306-4)
Supplement: Supplementary file 1 — (DOCX 18 kb) [file 11695_2019_4306_MOESM1_ESM.docx]

**Table S1**  **Characteristics of included studies**

| **Author** | **Year** | **Country** | **Study type** | | **Sample size** | | **NOS** |
| --- | --- | --- | --- | --- | --- | --- | --- |
|  |  |  |  | **LRYGB** | | **LSG** |  |
| Du X | 2016 | China | R | 63 | | 63 | 7 |
| Zhang Y | 2014 | China | RCT | 32 | | 32 | / |
| Rondelli F | 2016 | Italy | R | 301 | | 280 | 6 |
| Kehagias | 2011 | Greece | RCT | 30 | | 30 | / |
| [El Chaar](mailto:Maher.Elchaar@sluhn.org) | 2015 | ＵＳ | R | 547 | | 338 | 7 |
| Lee WJ | 2015 | Taiwan | R | 519 | | 519 | 6 |
| Peterli | 2013/2017/2018 | Switzerland | RCT | 110 | | 107 | / |
| Perron | 2017 | Italy | P | 142 | | 162 | 7 |
| Yang J | 2015 | China | RCT | 32 | | 32 | / |
| Boza | 2012 | Chile | R | 786 | | 811 | 6 |
| Ignat | 2016 | France | RCT | 45 | | 55 | / |
| Chouillard | 2011 | France | R | 283 | | 836 | 6 |
| Dogan | 2015 | Netherlands | R | 200 | | 200 | 7 |
| Helmiö, Mika | 2014/2018 | Finland | RCT | 119 | | 121 | 6 |
| Benaiges | 2011 | Spain | P | 95 | | 45 | 6 |
| Vidal | 2013 | US | P | 135 | | 114 | 7 |
| BandarAlbeladi | 2013 | France | R | 36 | | 34 | 7 |
| Cutolo | 2012 | Italy | R | 16 | | 15 | 6 |
| Nickel | 2017 | Germany | P | 38 | | 18 | 5 |
| Gill | 2016 | Canada | P | 51 | | 51 | 6 |

**R：**[retrospective](javascript:;) [study](javascript:;)

**P：**[prospective](javascript:;) [study](javascript:;)

**RCT：**[randomized](javascript:;) [controlled](javascript:;) [trial](javascript:;)

**NOS：**Newcastle–Ottawa scale
