# Supplementary figures and images for: A Comprehensive Comparison of LRYGB and LSG in Obese Patients Including the Effects on QoL, Comorbidities, Weight Loss, and Complications: a Systematic Review and Meta-Analysis
Source: Obes Surg. 2019 Dec 13;30(3):819–27. doi: 10.1007/s11695-019-04306-4 (PMC7347514; doi:10.1007/s11695-019-04306-4)

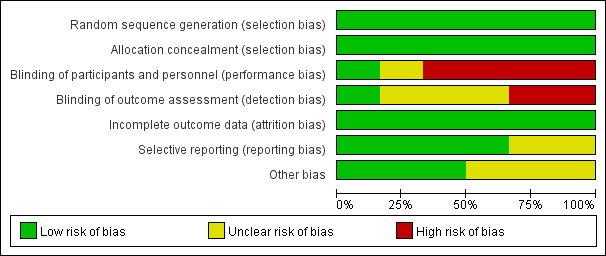

Supplement: Supplementary file 2 — (PNG 7 kb) [file 11695_2019_4306_MOESM2_ESM.png]
